# Effects of Light Quality and Photoperiod on Growth, Dry Matter Production and Yield of Ginger

**DOI:** 10.3390/plants14060953

**Published:** 2025-03-18

**Authors:** Haodan Zhang, Xingyue Li, Tao Han, Qin Huang, Junlan Liu, Ailin Tian, Linyu Liu, Guoqing Sun, Ling Dong, Hanyu Wang, Xintong Xie, Siyu Peng, Qiang Li, Honglei Li

**Affiliations:** 1 Chongqing Key Laboratory for Germplasm Innovation for Special Aromatic Spice Plants, Institute of Special Plants, College of Smart Agriculture, Chongqing University of Arts and Sciences, Chongqing 402160, China; 17723937896@163.com (H.Z.); 15023257221@163.com (X.L.); 15736171663@163.com (Q.H.); 15998916366@163.com (J.L.); 13114059319@163.com (A.T.); cqwuliulinyu@163.com (L.L.); 18325270389@163.com (G.S.); 17823794585@163.com (L.D.); 15023422200@163.com (H.W.); 13251377613@163.com (X.X.); 18581234479@163.com (S.P.); 2 College of Materials and New Energy, Chongqing University of Science and Technology, Chongqing 401331, China; hantao@cqust.edu.cn

**Keywords:** blue light, photoperiod, ginger, phenotypic characteristics, dry matter, yield

## Abstract

We investigated the effects of light quality and photoperiod on the phenotypic characteristics, dry matter production, and yield of ginger under three light quality ratios (A1: blue light: white light = 1:4; A2: blue light: white light = 1:1; A3: pure white light) and two photoperiod conditions (B1: 12/12 h·d^−1^; B2: 16/8 h·d^−1^). The results demonstrated that blue light treatment significantly reduced plant height and the dry matter distribution ratio of stems and sheaths. In contrast, stem diameter, tiller number, leaf area, theoretical biomass (TBY), maximum accumulation rate (*V*_max_), average accumulation rate (*V*_aver_), time point of maximum accumulation (*T*_max_), rapid growth period (DRGP), dry matter distribution ratio of leaves, roots, and rhizomes, number of rhizomes per plant, average rhizome weight, and yield all significantly increased with an increasing blue light ratio. Principal component analysis revealed distinct phenotypic traits, dry matter production characteristics, and yield-related traits under different blue light treatments. Blue light promoted tillering and increased stem thickness, which are key mechanisms for enhancing ginger yield. Additionally, prolonged photoperiods significantly increased plant height, stem diameter, branch number, leaf area, and biomass, while promoting the redistribution of photosynthetic products from leaves to rhizomes and increasing the proportion of dry matter allocated to rhizomes, thereby boosting ginger yield. These findings provide valuable insights into optimizing light conditions for ginger cultivation, highlighting the importance of a balanced blue-to-white light ratio and extended photoperiods in improving ginger growth and productivity.

## 1. Introduction

Ginger (*Zingiber officinal* Roscoe) is a perennial herb belonging to the Zingiberaceae family. Its rhizomes are rich in vitamins, minerals, proteins, and dietary fibers, have significant nutritional and medicinal value, and are widely planted worldwide [1,2]. With a large cultivated area and total global output China is the leading producer and exporter of ginger worldwide [3], accounting for approximately 70% of the total global trade. It is one of the most important characteristic vegetables for China’s exports. In addition, ginger is a characteristic industry in the ‘13th Five-Year Plan’ for agricultural planning in Southwest China and a crucial economic carrier for farmers in hilly and mountainous areas to eliminate poverty and increase income [4,5].

Light is one of the most crucial environmental factors in plant growth and development and has a wide range of regulatory effects on plant morphogenesis, physiological metabolism, growth and development, and yield formation [6,7]. Light quality can trigger light signal receptors in plants and affect their hormone contents and enzyme activities, which in turn affect their synthesis, metabolism, growth, and development [8]. As an inducing factor of seasonal dormancy, photoperiod affects the length of time for leaves to absorb and accumulate photosynthetically active radiation and significantly affects material production. Therefore, both the light quality and photoperiod have critical regulatory effects on plant growth and development [9]. Numerous studies have shown that blue light is one of the most important light sources in the visible spectrum, owing to its effect on plant growth and development [10]. Blue light improves the formation of plant roots, promotes the growth of rice [11], tobacco [12], tomato [13] and other plants, and increase root activity. It can also control the plant height of lettuce [14], cabbage [15], gourd [16] and other plants, increase the stem diameter, and increase stomatal opening and quantum yield to improve the photosynthetic capacity per unit leaf area, increase plant biomass, and ultimately significantly improve plant yield and quality [17]. Prolonging illumination time can increase plant biomass, improve nutritional quality, and promote plant growth and development. It can increase the biomass of lettuce, celery, and spinach [18] and expand the leaf area of zucchini [19] and melon [20].

Previous studies have focused on the effects on crops, but there is a lack of systematic research on the effects of blue light and photoperiod on the growth and yield formation of ginger. As an important economic crop, the light regulation mechanism of dry matter accumulation and distribution of ginger has not been systematically studied. In summary, blue light plays a vital role in regulating the growth and development of plants; however, studies on the effect of blue light on the growth and yield of ginger is still relatively rare. Ginger thrives in shade and requires artificial shading in the summer, with greenhouse conditions providing an ideal light environment [21,22]. Therefore, we used LED light sources to set different light qualities and photoperiods and indoor potted plants to investigate the effects of light quality and photoperiod on the phenotypic characteristics, dry matter production characteristics, and yield of ginger and provide a theoretical basis and technical support for the adaptation and regulation mechanism of the ginger light environment. If validated in field experiments, this approach could be integrated into precision agriculture frameworks.

## 2. Materials and Methods

### 2.1. Test Materials

The ginger variety tested was Yujiang No. 1, the primary cultivar in Chongqing, which was independently selected by this unit. The artificial light source LED lamp is T8 2835, and the light quality ratio is composed of the number ratio of different color beads. The tested soil was a typical purple soil from Southwest China. The basic soil nutrients were soil organic matter content 23.2 g kg^−1^, alkali hydrolysis nitrogen 39.7 mg kg^−1^, available phosphorus 108.7 mg kg^−1^, available potassium 298.5 mg kg^−1^, total nitrogen 1.4 g kg^−1^, total phosphorus 15.8 g kg^−1^, and total potassium 1.7 g kg^−1^, pH 7.03.

### 2.2. Experimental Design

The experiment was conducted in the artificial climate greenhouse of Chongqing University of Arts and Sciences (29°21′ N, 105°54′ E) from 2022 to 2023. Before the start of the experiment, the initial plant height, stem diameter, and leaf number of all ginger plants were measured to ensure that the status of each group at baseline was consistent. A two-factor randomized block experimental design was used. Based on the consistency of Factor A light quality and light intensity, three light quality ratios (A1: blue light: white light = 1:4; A2: blue light: white light = 1:1; A3: pure white light) and Factor B for two photoperiods (B1:12/12 h d^−1^; B2:16/8 h d^−1^). In this experiment, the ‘white light’ was provided by a (Shenzhen Yuxin Ou Technology Co., Ltd., Shenzhen, China) LED lamp with a spectrum range of 380–760 nm. A random number table was used to randomly assign all ginger plants to six treatment groups, with three replicates per treatment and 50 pots per replicate. Each pot was a biological replicate to ensure that there were no significant differences in plant height, stem diameter, and leaf number among the same groups at baseline. Each treatment was carried out in a separate culture chamber. The position of each pot was randomly changed every day, and the environmental conditions of the culture chamber were corrected every 3 days to ensure that the environmental factors of each pot plant were consistent except for the treatment conditions. The photoperiod was regulated using a timer, and the illumination of the B1 and B2 treatments was 7:00–19:00 and 5:00–21:00, respectively. Ginger seedlings (one per bag) were planted in seedling bags of 50 cm in height and 25 cm in diameter and placed in a steel-framed incubation shed with a height of 1.5 m and a width of 1 m. The perimeter of the shed was shaded with an impermeable black cloth to avoid interference with the light environment between treatments. The distance from the light source to the top of the ginger was adjusted to 30 cm so that the light intensity at the top of the ginger was 100 μmol m^−2^ s^−1^. The temperature in the greenhouse was 27 ± 1 °C, and the relative humidity was controlled at 55–60%. Although efforts were made to ensure the stability of environmental conditions throughout the experiment, some fluctuations that could not be eliminated may still have occurred. To minimize the impact of these factors, environmental conditions were monitored in real time during the experiment. The amount of fertilizer applied to each plant was calculated based on the application rate (kg/ha) used in the local high-yield demonstration field, scaled to pot surface area. The amounts of nitrogen (N), phosphorus (P_2_O_5_), and potassium (K_2_O) were 400 kg ha^−1^, 200 kg ha^−1^, and 150 kg ha^−1^, respectively. The P_2_O_5_ and K_2_O fertilizers were applied as basal fertilizers before sowing, and N fertilizers were applied in equal amounts before sowing and during tuber expansion.

### 2.3. Measurement of Indicators

#### 2.3.1. Morphological Indicators

Ten consecutive plants with uniform growth were selected from each plot at the mature stage of ginger, and the number of tillers, plant height, stem diameter, and leaf area were determined. The number of tillers was determined using a manual counting method. Only tillers with a height exceeding 3 cm from soil surface were counted. Plant height was measured from the base of the main stem to the highest point on the leaf using a ruler. Stem diameter was measured using a Vernier caliper, and the transverse and longitudinal diameters of the main stem at a height of 3 cm were measured and averaged. Leaf area was measured using the length-width coefficient method. The lengths and widths of all leaves were measured and calculated using the following formula: Leaf area = leaf length × leaf width × 0.75 [23].

#### 2.3.2. Physical Production Characteristics

Samples were taken at comparable developmental stages across both years, corresponding to 70, 100, 130, 160, and 195 days after seedling emergence in 2022, and 75, 105, 135, 165, and 197 days in 2023. Five plants with uniform growth were selected from each replicate of each treatment, and were divided into the roots, rhizomes, stem sheaths, and leaves. The samples were heated at 105 °C for 30 min to inactivate biological processes and then dried at 80 °C until constant weight. The biomass and dry matter distribution of each organ were then determined.

The theoretical biomass (*M_D_*), maximum accumulation rate (*V_mD_*), average accumulation rate (*V_aD_*), time point of the maximum accumulation rate (*T*_0*D*_), accumulation acceleration point (*T*_1*D*_), accumulation deceleration point (*T*_2*D*_), and rapid accumulation period (*T*_2*D*_ − *T*_1*D*_) were calculated using logistic equations based on Shi et al [24]. The formulas for calculating each parameter are as follows:

$$y=\frac{M_{D}}{1+{aEXP}^{-bx}}$$

$$V_{mD}=\frac{Mb}{4}$$

$$V_{aD}=\frac{M}{Growth\;period}$$

$$T_{0}=\frac{\sqrt{a}}{b}$$

$$T_{1D}=\frac{lna-1.317}{b}$$

$$T_{2D}=\frac{lna+1.317}{b}$$

where *x* is the number of days after emergence; *y* is the dry matter accumulation per plant; and a, b, and EXP are the equation coefficients.

#### 2.3.3. Yield and Composition

Ten pots per treatment and per replicate were assessed at maturity to determine the number of ginger rhizomes per plant, average rhizome weight, and total yield. The yield of ginger was determined using fresh weight.

### 2.4. Data Analysis

Data were processed using Excel 2010 and statistically analyzed using SPSS 19.0; statistical significance (*p* < 0.05) was determined using the least significant difference (LSD) method. Logistic growth equations were fitted using Origin 8, and GraphPad Prism 5.0 was used for data visualization. All continuous variables were evaluated for normality by Shapiro–Wilk test and Q-Q plot. If the data deviate from the normal distribution, the Box–Cox transformation is used for correction. The homogeneity of variance between groups was verified by Levene test. If the variance was not homogeneous (*p* < 0.05), a robust Welch’s ANOVA was used instead of the traditional ANOVA.

## 3. Results and Analysis

### 3.1. Effects of Light Quality and Photoperiod on Growth Characteristics of Ginger

**Figure 1:**
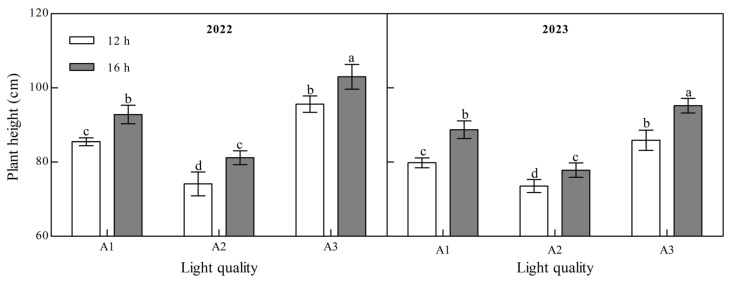
Effects of light quality and photoperiod on plant height of ginger. Note: The error bar represents the standard deviation. Different lowercase letters indicate that there are significant differences between different treatments (*p* < 0.05).

Light quality and photoperiod significantly affected ginger plant height at maturity (Figure 1, Appendix A). The plant height of ginger at maturity decreased with an increase in the blue light ratio (A2: blue:white = 1:1) compared to treatments with lower (A1: blue:white = 1:2) or higher (A3: blue:white = 2:1) blue light proportions over the two years. A2 was 13.27% and 22.46% lower than A1 and A3 at 12 h and 12.49% and 21.15% lower at 16 h in 2022. It was 7.87% and 14.37% and 12.22% and 18.21% lower in 2023, indicating that increasing the proportion of blue light could effectively control the plant height of ginger. The plant height of ginger under the B2 photoperiod was significantly higher than that under the B1 photoperiod. A1, A2, and A3 increased by 8.57%, 9.54%, and 7.73%, respectively, in 2022 and 11.11%, 5.87%, and 10.84%, respectively, in 2023, indicating that the extension of light exposure could increase the plant height of ginger.

**Figure 2:**
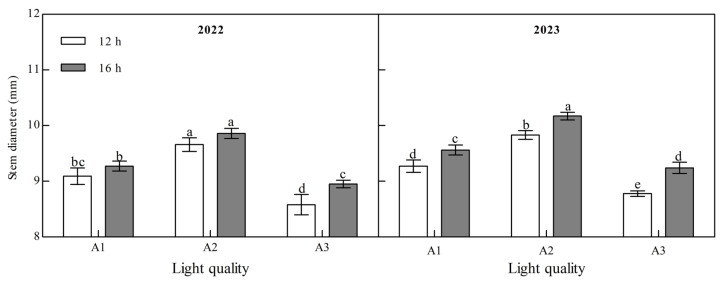
Effects of light quality and photoperiod on stem diameter of ginger. Note: The error bar represents the standard deviation. Different lowercase letters indicate that there are significant differences between different treatments (*p* < 0.05).

Light quality and photoperiod significantly affected the stem diameter of ginger at maturity (Figure 2, Appendix A). The stem diameter of mature ginger increased with an increase in the blue light ratio over two years. Under the two photoperiod treatments over the two years, the stem diameter of the A2 treatment was the largest and that of the A3 treatment was the smallest. A2 was 6.31% and 12.63% higher than A1 and A3 at 12 h and 6.29% and 10.09% higher at 16 h in 2022. It was 6.04% and 12.00% and 6.38% and 10.10% higher in 2023, indicating that increasing the proportion of blue light could effectively increase the stem diameter of ginger. The stem diameter of the ginger at the maturity stage was significantly higher under B2 than under B1 in the two years; A1, A2, and A3 were 2.05%, 2.04%, and 4.39%, respectively, higher in 2022, and 3.13%, 3.46%, and 5.24%, respectively, higher in 2023, indicating that increasing light duration can improve the stem diameter of ginger.

**Figure 3:**
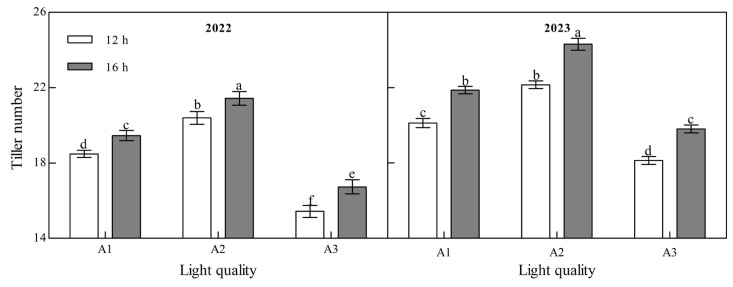
Effects of light quality and photoperiod on tiller number of ginger. Note: The error bar represents the standard deviation. Different lowercase letters indicate that there are significant differences between different treatments (*p* < 0.05).

Light quality and photoperiod had significant effect on the tiller number of ginger at maturity (Figure 3, Appendix A). The tiller number during the ginger maturation period increased with an increase in the blue light ratio over the two years. Under the two photoperiod treatments in the two years, the tiller number of the A2 treatment was the highest and that of the A3 treatment was the lowest. In 2022, the tiller number in A2 was 10.33% and 32.22% higher than that in A1 and A3 at 12 h, 10.11% and 27.97% higher than that in A1 and A3 at 16 h, 10.08% and 22.14%, 11.10%, and 22.73% higher than that in A1 and A3 in 2023, respectively, indicating that increasing the proportion of blue light can significantly increase the tiller number of ginger. In the two years of the B2 photoperiod, the number of tillers at the mature stage of ginger was significantly higher than that of the B1 photoperiod; A1, A2, and A3 increased by 5.27%, 5.05%, and 8.54%, respectively, in 2022 and by 8.71%, 9.72%, and 9.19%, respectively, in 2023, indicating that prolonging the light duration can promote the tillering of ginger and increase the number of tillers.

**Figure 4:**
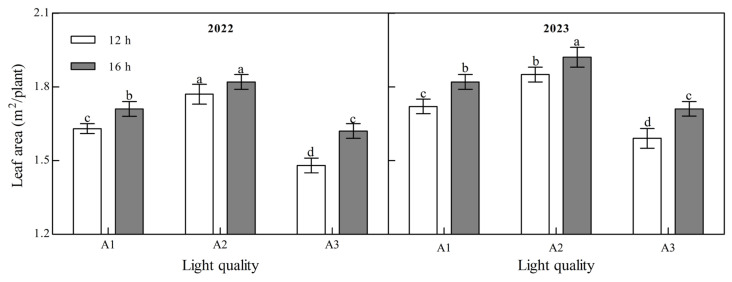
Effects of light quality and photoperiod on leaf area of ginger. Note: The error bar represents the standard deviation. Different lowercase letters indicate that there are significant differences between different treatments (*p* < 0.05).

Light quality and photoperiod had significant effects on the leaf area of ginger at maturity (Figure 4, Appendix A). The leaf area of mature ginger increased with an increase in the blue light ratio over the two years. Under the two photoperiod treatments over the two years, the leaf area of the A2 treatment was the largest and that of the A3 treatment was the smallest. In 2022, the leaf area in A2 was 8.79% and 19.55% higher than that in A1 and A3 at 12 h and 6.03% and 12.14% higher than those in A1 and A3 at 16 h, while it was 7.77%, 16.11%, 5.87%, and 12.48%, respectively, higher in 2023, indicating that increasing the proportion of blue light could significantly increase the leaf area of ginger. Leaf area at maturity of ginger in all light quality treatments in both years of B2 photoperiod was significantly higher than that of B1 photoperiod; 5.11%, 2.44%, and 9.21% higher in 2022 for A1, A2, and A3, respectively, and 5.83%, 3.96%, and 7.32% higher in 2023, suggesting that prolongation of light duration can improve leaf area of ginger.

### 3.2. Effects of Light Quality and Photoperiod on Dry Matter Production and Partitioning in Ginger

**Figure 5:**
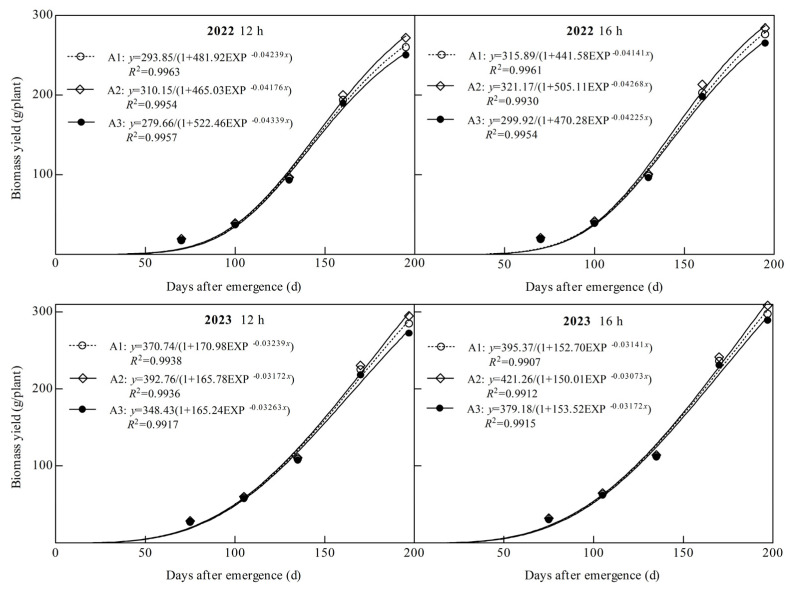
Logistic fitting curve of biomass under different treatments.

Dry matter production of ginger showed an “S” shaped growth curve throughout the reproductive period, and the coefficients of determination R2 of all logistic equations fitted in this study were above 0.99 (Figure 5). The theoretical biomass (TBY), maximum accumulation rate (*V_max_*), average rate (*V_aver_*), time point of maximum (*T_max_*), and rapid growth period (DRGP) of ginger were significantly affected by the light quality and photoperiod (Figure 5 and Table 1). TBY, *V_max_*, *V_aver_*, *T_max_* and DRGP increased significantly with an increasing percentage of blue light, with both photoperiodic treatments being the highest under the A2 treatment and the lowest under the A3 treatment in both years. In 2022, TBY, *V_max_*, *V_aver_*, *T_max_*, and DRGP in A2 at 12 h were 5.55%, 3.83%, 5.55%, 0.92%, and 1.51%, respectively, higher than those in A1 and 10.90%, 6.74%, 10.90%, 1.97%, and 3.90%, respectively, higher than those in A3. At 16 h, it was 1.67%, 4.59%, 1.67%, 0.87%, and 2.05%, respectively, higher than A1 and 7.08%, 8.17%, 7.08%, 1.01%, and 3.09%, respectively, higher than A3. At 12 h in 2023, A2 was 5.94%, 3.61%, 5.94%, 1.50%, and 2.11%, respectively, higher than A1 and 12.72%, 9.58%, 12.72%, 2.93%, and 2.87%, respectively, higher than A3. At 16 h, it was 6.55%, 4.07%, 6.55%, 1.85% and 2.21% higher than A1, 11.10%, 7.63%, 11.10%, 2.75%, and 3.22%, respectively, higher than A3. The results showed that increasing the proportion of blue light significantly increased the maximum and average accumulation rates of dry matter, delayed the time point of the maximum accumulation rate of dry matter, prolonged the rapid growth period of dry matter, and significantly increased the theoretical biomass of ginger.

Ginger TBY, *V_max_*, *V_aver_*, *T_max_*, and DRGP were significantly higher in all light quality treatments in both years of B2 photoperiod than in B1 photoperiod and were 7.50%, 4.99%, 15.30%, 0.08%, and 0.33%, respectively, higher in 2022 under A1 treatment and 3.55%, 5.83%, 11.06%, 0.02%, and 0.87%, higher in A2. It was 7.25%, 4.43%, 15.03%, 0.97%, and 1.66%, respectively, higher in A3. In 2023, A1 was 6.65%, 3.42%, 14.38%, 0.85%, and 3.12%, respectively, higher; 7.26%, 3.91%, 15.04%, 1.20%, and 3.22%, higher in A2; and 8.83%, 5.79%, 16.72%, 1.39%, and 2.87%, respectively, in A3. This indicated that prolonging the light duration could increase the maximum dry matter accumulation rate and the average accumulation rate of ginger, delay the time point of the maximum dry matter accumulation rate, and prolong the period of rapid growth of dry matter, which significantly increased the theoretical biomass of ginger.

**Figure 6:**
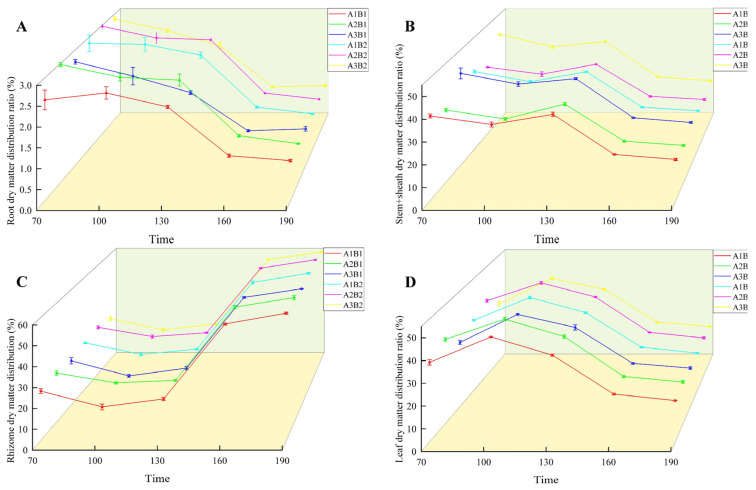
Differences in ginger dry matter distribution ratio in different organs (2022). Note: root dry matter distribution ration (**A**); stem+sheath dry matter distribution (**B**); rhizome dry matter distribution (**C**); leaf dry matter distribution ratio (**D**); time, days after sowing (days).

**Figure 7:**
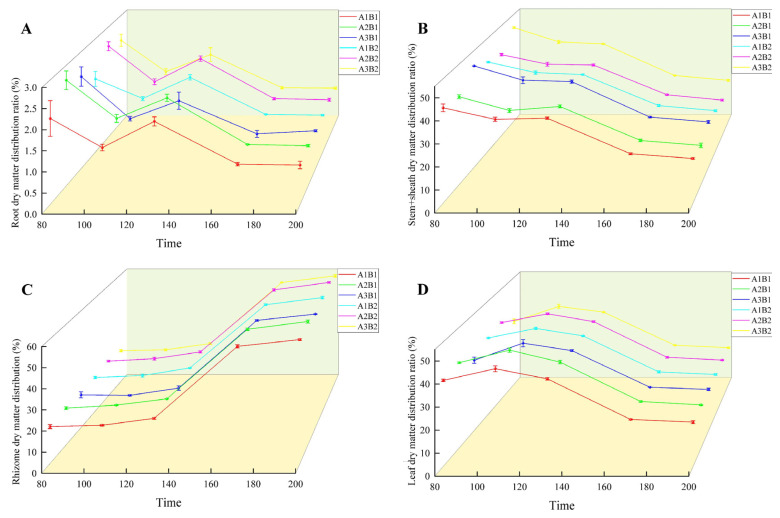
Difference in ginger dry matter distribution ratio in different organs (2023). Note: root dry matter distribution ration (**A**); stem+sheath dry matter distribution (**B**); rhizome dry matter distribution (**C**); leaf dry matter distribution ratio (**D**); time, days after sowing (days).

During the growth of ginger, the distribution ratio of dry matter in the root and stem sheaths decreased gradually. In the two years, the treatment was highest in the early growth period and lowest in the mature period (Figure 6 and Figure 7). The proportion of dry matter distribution in the leaves first increased and then decreased with the growth process, peaking at 100 d and being the lowest at maturity. In contrast, the proportion of dry matter distribution in the rhizomes increased with the growth process and peaked at maturity. Light quality and photoperiod significantly affected the dry matter distribution ratio of ginger organs. The distribution ratio of dry matter in the leaves, roots, and rhizomes of ginger increased gradually in each period with an increase in the proportion of blue light, being the highest in the A2 treatment and the lowest in the A3 treatment. The dry matter distribution ratios of the leaves, roots, and rhizomes in A2 were 2.12%, 0.44%, 0.81%, 1.19%, 0.06%, 2.05%, 0.94%, 0.18%, 1.07%, 0.37%, 0.05%, 1.21%, 0.32%, 0.02%, and 1.25% higher, respectively, than those in A1. The dry matter distribution ratios of the stems and sheaths in A2 were lower than those in A1 by 3.36%, 3.30%, 2.20%, 1.63% and 1.60% at 70, 100, 130, 160, and 195 days after emergence, respectively. The dry matter distribution ratios of leaves, roots, and rhizomes in A2 were 6.96%, 0.21%, 1.86%, 3.52%, 0.23%, 3.56%, 1.98%, 0.47%, 2.43%, 1.07%, 0.18%, 2.09%, 0.65%, 0.03%, and 2.51% higher than those in A3, respectively, and the dry matter distribution ratios of stems and sheaths were 9.03%, 7.32%, 4.88%, 3.33%, and 3.19% lower than those in A3, respectively, indicating that increasing the proportion of blue light could effectively increase the dry matter distribution ratio of leaves, roots, and rhizomes of ginger at each stage, and reduce the dry matter distribution ratio of stems and sheaths.

In terms of photoperiod, the proportion of leaf dry matter allocation was higher at all sampling points in the 12 h treatment than in the 16 h treatment, while the proportion of tuber dry matter allocation was lower than in the 16 h treatment. Proportions of leaf dry matter allocation were higher than those of the 16 h treatment by 0.95%, 1.43%, 0.58%, 0.31%, and 0.35% at each sampling point, while the proportions of tuber dry matter allocation were lower than those of the 16 h treatment by 0.45%, 1.07%, 1.10%, 0.33%, and 0.38%, indicating that prolonging the light duration can effectively reduce the dry matter allocation proportion of ginger leaves while increasing the dry matter allocation.

### 3.3. Effect of Light Quality and Photoperiod on Ginger Yield and Its Composition

Light quality and photoperiod significantly affected the number of ginger rhizomes per plant, the average weight of ginger rhizomes, and ginger yield (Table 2). The number of ginger rhizomes per plant, average rhizome weight, and yield of ginger increased with the proportion of blue light in both years. The number of ginger rhizomes per plant, average ginger rhizome weight, and yield of ginger were the highest in the A2 treatment and lowest in the A3 treatment under both photoperiodic treatments in both years. In 2022, the number of ginger rhizomes per plant, average ginger rhizome weight, and yield in A2 after 12 h were 8.67%, 11.59%, and 8.55 higher than those in A1 and 14.06%, 27.36%, and 18.76% higher than those in A3. At 16 h, it was 7.28%, 10.64%, and 7.73% higher than that in A1 and 11.88%, 22.84%, and 17.60% higher than that in A3. In 2023, it was 6.17%, 5.83% and 8.12% higher than that in A1 and 9.70%, 13.78% and 15.01% higher than that in A3. At 16 h, they were 7.61%, 5.83%, and 9.49% higher than that in A1 and 11.56%, 12.39%, and 15.70% higher than that in A3. This indicated that increasing the proportion of blue light can significantly increase the number of ginger rhizomes per plant, increase the average weight of ginger rhizomes, and ultimately increase the yield of ginger. The number of ginger rhizomes per plant, average weight of ginger rhizomes, and yield of ginger treated with different light qualities under the B2 photoperiod were significantly higher than those under the B1 photoperiod over the two years. Under the longer photoperiod treatment (B2: 16 h light) compared to shorter photoperiod (B1: 12 h light), the average number of ginger rhizomes per plant, average rhizome weight, and total yield were 4.98%, 6.10%, and 4.49% higher, respectively, in 2022 and 4.08%, 6.14%, and 3.44% higher than those in B1 in 2023, respectively. The results showed that prolonging the light period could increase the number of ginger rhizomes per plant and the average weight of ginger rhizomes, thereby increasing the yield.

**Figure 8:**
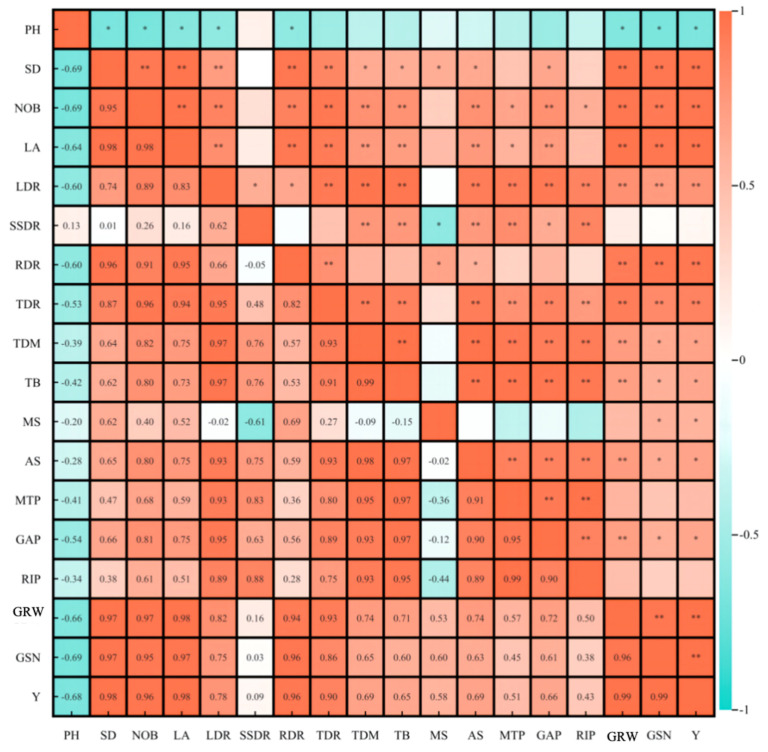
Correlation between phenotypic characteristics, dry matter accumulation and distribution, yield and yield components of ginger. Note: ** and * indicated significant difference at *p* ≤ 0.01 and *p* ≤ 0.05.

The phenotypic characteristics, dry matter accumulation, and distribution of ginger had significant effects on the yield and its components (Figure 8). Ginger rhizome weight was significantly and positively correlated with stem diameter (0.97), number of branches (0.97), leaf area (0.98), root dry matter distribution ratio (0.94), tuber dry matter distribution ratio (0.93), leaf dry matter distribution ratio (0.82) and significantly negatively correlated with plant height (−0.66). Ginger rhizomes per plant were significantly positively correlated with stem diameter (0.97), number of branches (0.95), leaf area (0.97), root dry matter distribution ratio (0.94), and tuber dry matter. Yield was significantly positively correlated with stem diameter (0.98), number of branches (0.96), leaf area (0.98), root dry matter distribution ratio (0.96), tuber dry matter distribution ratio (0.90), ginger rhizome weight (0.99), and ginger rhizomes per plant (0.99). It was negatively correlated with plant height (−0.68), indicating that light and photoperiod regulated the dry matter accumulation and distribution of ginger by affecting the phenotypic characteristics of ginger and ultimately affected the yield and composition of ginger.

**Figure 9:**
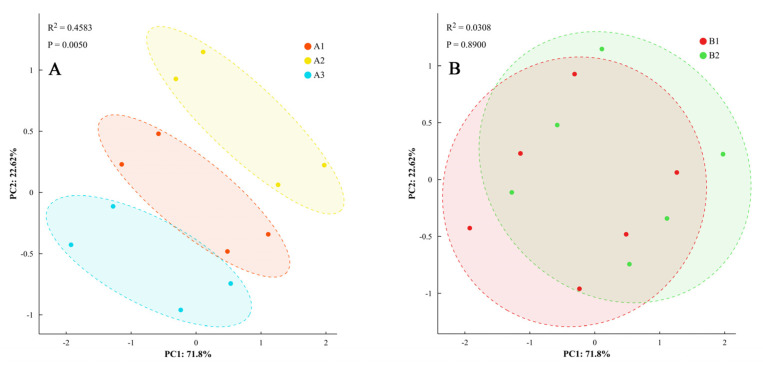
Principal component analysis of phenotypic traits. Note: dry matter accumulation and distribution and yield of ginger with different light quality (**A**) and photoperiod (**B**).

Principal component analysis of the differences in phenotypic characteristics was performed, with components having eigenvalues >1 retained (explaining 78.3% of total variance). Analysis revealed that dry matter accumulation, distribution characteristics, and ginger yield under different light quality and photoperiod treatments over two years indicated that the differences in phenotypic characteristics, dry matter accumulation and distribution characteristics, and yield of ginger under different light quality treatments were significant (Figure 9). It could be classified into three categories, whereas the effects of different photoperiods on phenotypic characteristics, dry matter accumulation, distribution, and yield showed statistical differences (*p* < 0.05) but with smaller magnitudes (4.1–6.1% difference) compared to light quality effects (7.9–22.5% difference).

## 4. Discussion

### 4.1. Effect of Light Quality and Photoperiod on Phenotypic Characteristics of Ginger

Light is one of the most crucial environmental factors for plant growth and development, both as an energy source for plant photosynthesis and as an environmental signal to regulate plant morphogenesis [25]. The influence of light on plant growth is mainly reflected by three aspects: light quality, light intensity, and photoperiod. Under certain light intensities, plants respond biologically to changes in the light quality and photoperiod [26]. Blue light can inhibit cell elongation, which is beneficial for controlling the height of lettuce [27], cabbage [28], and gourd [16]. This growth pattern often redirects resources to lateral expansion, thereby increasing stem diameter in these plants and potentially in ginger as well. Liu et al. [29] also found that blue light could increase the length and width of lettuce leaves, increase the leaf area, and improve morphological indices. The results of this experiment showed that the stem diameter, tiller number, and leaf area of ginger significantly increased after blue light treatment. In contrast, the plant height significantly decreased, and the amplitude increased with an increase in the blue light ratio. Under the A2 treatment (blue light: white light = 1:1), the stem diameter, tiller number, and leaf area of ginger were the largest, whereas the plant height was the lowest, which was consistent with the results of Jiang et al. [30] for other plants. Blue light not only inhibits the longitudinal elongation of cells, reduces plant height, and increases stem diameter but also increases the thickness of the leaf epidermis and the length and width of palisade cells, increasing the area of leaf guard cells, stomatal density, and stomatal conductance, thereby increasing leaf area and regulating photosynthesis. With the extension of illumination time, plant height, stem diameter, tiller number, and leaf area of ginger increased significantly, which was consistent with the results of Xie et al. [31]. The extension of the illumination time was accompanied by an increase in photosynthesis time and the synthesis of sufficient organic matter, which accelerated the growth and biomass accumulation of plants. Therefore, plant height, stem diameter, tiller number, and leaf area of ginger increased significantly. However, a potential source of error is the inconsistency of light intensity, which may affect the repeatability of the experimental results. Future research can solve one of the problems by controlling the light intensity more accurately.

### 4.2. Effects of Light Quality and Photoperiod on Dry Matter Accumulation and Partitioning in Ginger

The accumulation and distribution of photosynthetic assimilates are the basis for plant growth and yield. Both light quality and photoperiod regulate plant dry matter accumulation and distribution [32]. Li [33] pointed out that a high blue light ratio can reduce the dry matter distribution ratio of the soybean stem sheath and petiole and increase the dry matter distribution ratio of the leaves and roots. Gu et al. [34] found that prolonging the photoperiod increased the relative growth rate of red sand seedlings, which in turn increased their biomass. The results of this experiment showed that ginger TBY, *V_max_*, *V_aver_*, *T_max_*, and DRGP significantly increased with an increase in the blue light ratio, which is consistent with the findings in tomato, lettuce [35], and blueberry [36]. An increase in the blue light ratio can improve the development of chloroplasts in leaves, promote the formation of chlorophyll, regulate the opening and closing of stomata, and affect plant dry matter accumulation. In addition, blue light inhibited stem elongation and reduced the proportion of dry matter distribution in the stem sheath, whereas the proportion of dry matter distribution in the leaves, roots, and rhizomes increased, which is consistent with the results of previous studies [33]. The increase in the leaf dry matter distribution ratio and leaf area is also a key reason for blue light increasing ginger dry matter production. With the extension of the photoperiod, the TBY, *V_max_*, *V_aver_*, *T_max_*, and DRGP of ginger significantly increased, which was consistent with the results of Zhang et al. [37], who found that a prolonged photoperiod promoted plant growth metabolism and physiological processes and consequently improved biomass accumulation. Prolonging the photoperiod increases the duration of photosynthesis in plant leaves, tilts the distribution of photosynthetic products to other organs, and decreases the photosynthetic products allocated to the leaves. Therefore, the proportion of dry matter distribution in ginger leaves decreased and that in rhizomes increased under B2 treatment, which was similar to the results of Wu et al. [38].

### 4.3. Relationships Between Phenotypic Characteristics, Dry Matter Accumulation, and Partitioning Characteristics and Yield of Ginger

The morphogenesis of plants directly affects their growth and development, which, in turn, affects their yield and quality. As the final product of plant photosynthesis, the accumulation and distribution characteristics of dry matter are the basis for the formation of yield and quality [39]. Dong et al. [40] indicated that the number of tillers and stem thickness of ginger indirectly regulate ginger yield, mainly by affecting the number of ginger rhizomes per plant and the average weight of ginger rhizomes, whereas plant height and dry matter accumulation directly regulate ginger yield. Li et al. [41] found that ginger yield was significantly positively correlated with stem diameter, stem fresh weight, plant height, and leaf fresh weight, whereas it was significantly and negatively correlated with rhizome dry matter content and tiller number. The results of this experiment showed that ginger yield was significantly and positively correlated with stem diameter, tiller number, root and tuber dry matter distribution ratio, tuber dry matter distribution ratio, and theoretical biomass, but significantly negatively correlated with plant height, which is inconsistent with the results of Li et al. [41]. The reason is that Li et al. [41] found that yield was significantly negatively correlated with branch number (−0.3680) and significantly positively correlated with plant height (0.6024), while our results showed that yield was significantly positively correlated with number of branches (0.96) and significantly negatively correlated with plant height (−0.68). The stem diameter and tiller number of ginger are originally contradictory. In the traditional test of ginger, the rhizome number increases, and the weight of a single rhizome decreases, resulting in a decrease in stem diameter. In this experiment, blue light treatment can promote the number of tillers, increase the growth of stem diameter, leaf area and biomass, increase the rhizome number and individual rhizome weight, and ultimately increase the yield. This was consistent with the results of Chang et al. [42], who reported that blue light improved potato dry matter accumulation, promoted early tuberization, increased tuberization number, inhibited plant height, and increased potato yield. The yield of ginger was significantly positively correlated with the number of ginger rhizomes per plant and the average weight of the ginger rhizomes, whereas the number of ginger rhizomes per plant was significantly negatively correlated with the average weight of the ginger rhizomes. The dynamic balance between the two determines the yield. At the same time, the number of ginger rhizomes per plant was significantly positively correlated with the number of tillers, and the average weight of ginger rhizomes was significantly positively correlated with stem diameter [40]. Blue light treatment promoted tillering and improved stem diameter in ginger. These morphological changes were positively correlated with increased photosynthetic surface area and enhanced structural support, ultimately leading to higher yields. Principal component analysis revealed that the first two components explained 78.3% of the total variance, with clear separation of treatment groups (ANOSIM, R = 0.82, *p* < 0.001), indicating significant differences in phenotypic characteristics, production traits, and yield components under different blue light treatments.

In the previous study, only the above-ground and underground parts of ginger, potato, and other crops were tested. This experiment systematically studied the regulation of blue light on the growth and development of ginger for the first time, especially the comprehensive effect of blue light on the above-ground part (stem diameter, plant height, leaf area) and underground part (tuber number, tuber weight, dry matter distribution) of ginger. By clarifying that blue light can improve the yield of ginger by regulating the morphogenesis of the above-ground part and the distribution of dry matter in the underground part, this experiment provides theoretical support for the efficient cultivation of ginger. This discovery not only provides a new cultivation strategy for increasing ginger yield but also provides a potential solution to the challenges of agricultural production caused by global climate change.

## 5. Conclusions

Light quality and photoperiod affected the morphogenesis of ginger (plant height, stem diameter, tiller number, and leaf area) and the dry matter production of ginger (TBY, *V_max_*, *V_aver_*, *T_max_*, and DRGP), thereby affecting the yield and yield components. Blue light treatment inhibited the elongation of ginger stems, significantly reduced plant height, and significantly increased stem diameter, tiller number, leaf area, biomass, leaf and tuber dry matter distribution ratio, and yield; the amplitude increased with an increase in blue light ratio. Principal component analysis showed that the phenotypic characteristics, dry matter production characteristics, and yield traits of ginger differed significantly under different blue light treatments. Blue light promoted tillering and increased the stem diameter of ginger, which is an essential mechanism for increasing ginger yield. With regard to photoperiod, extending the light period from 12 h to 16 h significantly increased plant height (4.1%) and stem diameter (6.1%), and it also increased the distribution ratio of tuber dry matter, resulting in higher tuber dry matter distribution and yield. In summary, our results demonstrate that the optimal growth conditions for ginger combine a balanced blue/white light ratio of 1:1 with an extended photoperiod of 16 h, resulting in improved morphological traits and enhanced yield components. However, its detailed physiological and molecular mechanisms need to be further clarified. Future research can be carried out from both physiological and molecular mechanisms to further explore the effect of blue light on the growth of ginger and apply it to actual production.

## Figures and Tables

**Table 1:** Dry matter accumulation characteristics of ginger under different treatments.

Years	Treatments	TBY (g Plant^−1^)	*V*_max_/(g d^−1^)	*V*_aver_/(g d^−1^)	*T*_max_/d	DRGP
2022	A1B1	293.85 e	3.11 c	1.42 c	145.74 b	62.14 b
	A2B1	310.15 c	3.24 b	1.50 b	147.08 a	63.07 a
	A3B1	279.66 f	3.03 d	1.35 d	144.24 c	60.71 d
	Mean	294.55 B	3.13 B	1.42 B	145.69 B	61.97 B
	A1B2	315.89 b	3.27 b	1.64 a	145.85 b	62.34 b
	A2B2	321.17 a	3.43 a	1.66 a	147.11 a	63.62 a
	A3B2	299.92 e	3.17 c	1.55 b	145.64 b	61.72 c
	Mean	312.33 A	3.29 A	1.62 A	146.20 A	62.56 A
2023	A1B1	370.74 c	3.00 c	1.79 d	158.74 c	81.32 c
	A2B1	392.76 b	3.11 b	1.90 c	161.12 b	83.04 b
	A3B1	348.43 d	2.84 d	1.68 e	156.52 d	80.72 c
	Mean	370.64 B	2.99 B	1.79 B	158.79 B	81.69 B
	A1B2	395.37 b	3.10 b	2.05 b	160.09 b	83.86 b
	A2B2	421.26 a	3.24 a	2.18 a	163.06 a	85.71 a
	A3B2	379.18 c	3.01 c	1.96 bc	158.70 c	83.04 b
	Mean	398.61 A	3.12 A	2.07 A	160.61 A	84.20 A

Note: TBY: theoretical biomass yield; *V*_max_: maximum accumulation rate; *V*_aver_: average accumulation rate; *T*_max_: days to maximum accumulation rate; DRGP: duration of rapid growth period. Different lowercase letters indicate a significant difference among treatments; Different uppercase letters indicate a significant difference between photoperiods.

**Table 2:** Effects of light quality and photoperiod on ginger yield and yield components.

Treatments	Ginger Rhizomes Per Plant		Ginger Rhizome Weight (g)		Yield (g Plant^−1^)	
	2022	2023	2022	2023	2022	2023
A1B1	15.93 d	16.70 c	26.31 c	27.73 d	419.22 d	439.74 cd
A2B1	17.78 b	17.67 b	28.59 b	29.43 b	455.06 b	475.46 b
A3B1	13.96 f	15.53 d	25.06 d	26.83 e	383.17 f	413.41 e
Mean	15.89 B	16.63 B	26.65 B	28.00 B	419.15 B	442.87 B
A1B2	16.82 c	17.65 b	27.69 b	28.75 c	438.95 c	451.92 c
A2B2	18.61 a	18.68 a	29.70 a	30.94 a	472.89 a	494.79 a
A3B2	15.15 e	16.62 c	26.55 c	27.73 d	402.11 e	427.66 d
Mean	16.86 A	17.65 A	27.98 A	29.14 A	437.54 A	458.12 A
Source of variation						
Light quality (L)	203.97 **		170.71 **		83.34 **	
Photoperiod (P)	73.54 **		77.58 **		50.34 **	
Year (Y)	43.72 **		79.83 **		267.57 **	
L × P	0.40 ^ns^		0.07 ^ns^		0.56 ^ns^	
L × Y	14.70 **		0.80 ^ns^		1.82 ^ns^	
P × Y	0.05 ^ns^		0.44 ^ns^		0.11 ^ns^	
L × P × Y	0.12 ^ns^		1.10 ^ns^		0.31 ^ns^	

Note: ** indicated significant difference at *p* ≤ 0.01 and *p* ≤ 0.05, ns indicated that the difference being tested is not statistically significant. Different lowercase letters indicate a significant difference among treatments; Different uppercase letters indicate a significant difference between photoperiods.

## Data Availability

Data are contained within the article.

## Abbreviations

Y	yield
PH	plant height
SD	stem diameter
TB	theoretical biomass
LA	leaf area
MS	maximum accumulation speed
AS	average accumulation speed
MTP	days to maximum accumulation rate
GAP	growth acceleration point
RIP	duration of rapid growth period
GRW	ginger rhizome weight
GSN	ginger rhizomes per plant
NOB	
number of branches	
LDR	leaf dry matter distribution ratio
SSDR	stem sheath dry matter distribution ratio
RDR	root dry matter distribution ratio
TDR	tuber dry matter distribution ratio
TDM	total dry matter

## Appendix A

**Table A1:** ANOVA analysis on growth indexes of ginger under different light quality and photoperiod.

Factor	Plant Height		Stem Thick		Tiller Number		Leaf Area		Total Dry Matter	
	P	F	P	F	P	F	P	F	P	F
Y	1	46.95 **	1	47.51 **	1	700.39 **	1	86.76 **	1	242.20 **
Q	2	192.43 **	2	267.68 **	2	827.03 **	2	181.18 **	2	61.93 **
P	1	94.26 **	1	76.62 **	1	263.47 **	1	80.81 **	1	92.95 **
Y × Q	2	6.70 **	2	0	2	7.62 *	2	0.06	2	0.02 **
Y × P	1	0.02	1	2.44	1	16.91 **	1	0.19	1	0
Q × P	2	0.98	2	2.50	2	0.52	2	3.70 *	2	0.33
Y × Q × P	1	46.95	2	0.06	2	1.50	2	0.53	2	0.32

Note: Y: year; Q: quality of light; P: photoperiod. ** and * indicated significant difference at the *p* ≤ 0.01 and *p* ≤ 0.05 level.
